# The Bone Black Pigment Identification by Noninvasive, In Situ Infrared Reflection Spectroscopy

**DOI:** 10.1155/2018/6595643

**Published:** 2018-03-15

**Authors:** Alessia Daveri, Marco Malagodi, Manuela Vagnini

**Affiliations:** ^1^Laboratorio di Diagnostica per i Beni Culturali di Spoleto, Rocca Albornoziana, Piazza B. Campello 2, 06049 Spoleto, Italy; ^2^Laboratorio Arvedi di Diagnostica Non Invasiva, Università di Pavia, via Bell'Aspa 3, 26100 Cremona, Italy

## Abstract

Two real case studies, an oil painting on woven paper and a cycle of mural paintings, have been presented to validate the use of infrared reflection spectroscopy as suitable technique for the identification of bone black pigment. By the use of the sharp weak band at 2013 cm^−1^, it has been possible to distinguish animal carbon-based blacks by a noninvasive method. Finally, an attempt for an eventual assignment for the widely used sharp band at 2013 cm^−1^ is discussed.

## 1. Introduction

Over the centuries, carbon-based black pigments have represented a considerable group of pigments used in different types of works of art since ancient times. According to the convention proposed by Winter [1], the carbon-based pigments can be classified according to the starting materials (plants, animals, and minerals) and to the manufacturing processes (flame carbons, chars, and cokes) [2].

Several studies have been devoted to the molecular characterization of carbon-based black pigments by means of different destructive and microdestructive techniques such as Raman, SEM-EDX, XRD, ICP-AES, and FTIR [3–7]. Generally, the diagnostic difficulties which lie in the characterization of this pigment are due to the various origins and/or manufacturing processes related to (i) characterization of carbonaceous phase [8] and (ii) identification of noncarbon constituents. Regarding this last point, the most obvious example of noncarbon constituents is the inorganic materials in animal bone pigments. According to Winter's classification, these pigments belong to the coke group, being produced from plastic precursor like the collagen [1]. Indeed, in the bone black, the collagen forms a coke that is intimately mixed with hydroxyapatite Ca_5_(OH)(PO_4_)_3_, which consequently could be considered a clue for its identification.

Numerous studies have been published concerning bone black identification through the detection of phosphates by microdestructive techniques (SEM-EDS, micro-Raman, micro-FTIR, and micro-XRD) [3, 9, 10]. From these, it can be deduced that micro-Raman technique is not always able to distinguish the origin of those pigments because the detectable band at 960 cm^−1^ assignable to the symmetric stretching of phosphate is not always visible in bone blacks [9–11].

SEM-EDS study may reveal the nature of the bone black pigment both considering the elemental composition, namely, the combined detection of Ca and P, and the different morphologies that diverse sources and origin of carbon-based material show [1, 2]. In addition, elemental composition analysis may also distinguish between ivory and bone black on the basis of the higher content of magnesium in the ivory black-based pigment [3, 12].

XRD has proven to be a valuable tool for identifying the crystalline noncarbon constituent hydroxyapatite in the ivory and bone blacks [3].

Finally, by infrared spectroscopy, it is possible to distinguish animal carbon-based blacks thank to the presence of a sharp band at 2013 cm^−1^ in the infrared spectra of bone pigments [13, 14]. Although this band is generally used for identifying and distinguishing the bone black pigments from the others, its assignment is still uncertain.

In all the previous cited studies, the investigation was conducted by both destructive and microdestructive techniques and nondestructive techniques which generally imply the need for sampling. In recent years, the noninvasive approach has been preferred in order to preserve the integrity of artworks and to avoid their transportation. Among the portable techniques, FTIR in reflection mode has been already demonstrated a valuable tool for the noninvasive identification of both organic and inorganic materials (including bone black pigments) in work of arts [15, 16]. The main limitation of the technique is related to the possible distortion arising from the specular and diffuse components of the reflected light that may strongly alter the conventional appearance of the infrared spectra, thus making any interpretation difficult.

Therefore, powdered commercial bone black pigments were analyzed by infrared spectroscopy in transmission and reflection mode. An oil painting on woven paper and a mural painting were investigated by infrared reflection spectroscopy for the noninvasive identification of animal carbon black pigments. Finally, the bone black and hydroxyapatite spectra were compared in order to find a possible molecular assignment of the widely used infrared band at 2013 cm^−1^.

## 2. Materials and Methods

### 2.1. Bone Black Pigments and Model Painting Replicas

Ivory black pigments by Maimeri and Bresciani, lamp black by Zecchi, and hydroxyapatite by Carlo Erba were selected. The test panels were prepared applying the two commercial black pigments with an acrylic binder (primal), onto a commercial panel support primed with calcium carbonate and an acrylic medium. The pigment/binding medium ratio was 1 : 1 wt/wt.

### 2.2. Real Artworks

An oil painting on woven paper depicting a “drummer boy,” signed HD in the lower left and ascribable to the style of the French caricaturists of the late XIXs, belonging to a private collection, previously a restoration procedure, has been investigated by means of noninvasive technique.

A cycle of mural paintings, executed by Lo Spagna in 1526-1528 has been analyzed during a wide diagnostic campaign at the presbytery of St. Giacomo Church in Spoleto, Italy.

### 2.3. Portable Mid-Infrared Spectrophotometer

The portable mid-infrared spectrophotometer ALPHA-R Bruker Optics is equipped with a Globar radiation source, a modified Michelson interferometer (RockSolid™) and a DLaTGS detector. Its weight is about 7 kg, and its dimensions are 20 × 30 × 12 cm^3^. Exchangeable QuickSnap™ sampling modules have allowed us to perform transmission and external reflection measurements.

### 2.4. Sample Compartment Module

The universal sampling module is equipped with a transmission compartment with standard sample holder. The spectra have been recorded with 100 scans, a resolution of 4 cm^−1^ and an effective range of 7500–375 cm^−1^ in KBr pressed disks.

### 2.5. Module for External Reflection

The module for external reflection enables us to work with an angle of incidence of about 20°. It is equipped with an integrated video camera for the control and monitoring of the sampling area. The investigated sample area is about 28 mm^2^. The spectrum intensity has been defined as the pseudo absorbance as log (1/*R*). The background correction has been measured using the spectrum from a gold mirror surface. The spectra have been collected in the 7500–375 cm^−1^ range, with a resolution of 4 cm^−1^ and 186 scans.

### 2.6. Optical Microscopy

A preliminary evaluation of cross section and a study of the different layers were performed by a light-polarized microscope Olympus BX51TF, equipped with the Olympus TH4-200 lamp (visible light) and the Olympus U-RFL-T (UV light). The sessions were carried out in reflection mode at different magnifications, (10x, 20x, and 50x).

### 2.7. Scanning Electron Microscopy (SEM-EDS)

Scanning electron microscopy (SEM) images and energy-dispersive X-ray spectra (EDS) were collected by using a Tescan FE-SEM, MIRA XMU series equipped with EDAX spectrometer, at an accelerating voltage of 15–20 kV, and high vacuum. The sample surfaces were metalized with a coating of graphite by Cressington 208HR sputter.

## 3. Results and Discussion

**Figure 1:**
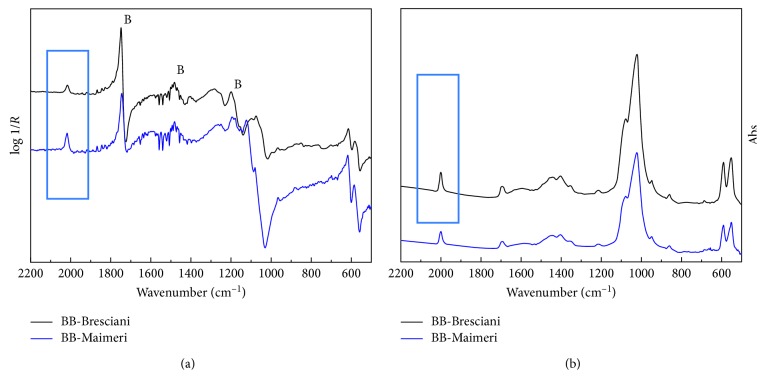
FTIR spectra in (a) reflection mode and (b) transmission mode of commercial animal black pigments and mockup, respectively. The acrylic binder (B) signals are highlighted.

The comparison of FTIR spectra in transmission and in reflection mode of the animal commercial black pigments as pure powder and applied onto panel is shown in Figure 1. The transmission infrared spectra show the characteristic phosphate group bands: the *ν*_3_ (PO_4_)^3−^ at 1087 and 1038 cm^−1^, the *ν*_1_ (PO_4_)^3−^ at 875 and 962 cm^−1^, the *ν*_4_ (PO_4_)^3−^ at 630, 604, and 567 cm^−1^, and the *ν*_2_ (PO_4_)^3−^ at 469 cm^−1^ (Figure 1). The identical features, together with the acrylic binder signals (highlighted with B, in Figure 1), are recognizable in the infrared spectra in reflection mode as derivative and/or reststrhalen bands that are strongly distorted by the surface reflection and difficult to use for diagnostic purposes [17]. All the FTIR spectra display also a weak-sharp band at 2013 cm^−1^. This not yet unsigned feature is not affected by the surface reflection, and it is diagnostic for the identification and distinction of the animal black pigments [13, 14].

This peculiar feature is easily detected in real artworks by means of infrared spectroscopy working in transmission, as a microdestructive technique [13, 14], and in reflection mode as noninvasive method directly on the painting surfaces. Some papers have already been published highlighting the effectiveness of this technique for detecting the presence of this band for the noninvasive and in situ identification of animal black on real works of art [15, 16].

Below are details concerning two studies carried out on different material types of artworks are reported: an oil painting on woven paper probably ascribable to *Honorè Daumier* and a mural painting by *Lo Spagna*.

### 3.1. Painting Oil on Woven Paper

**Figure 2:**
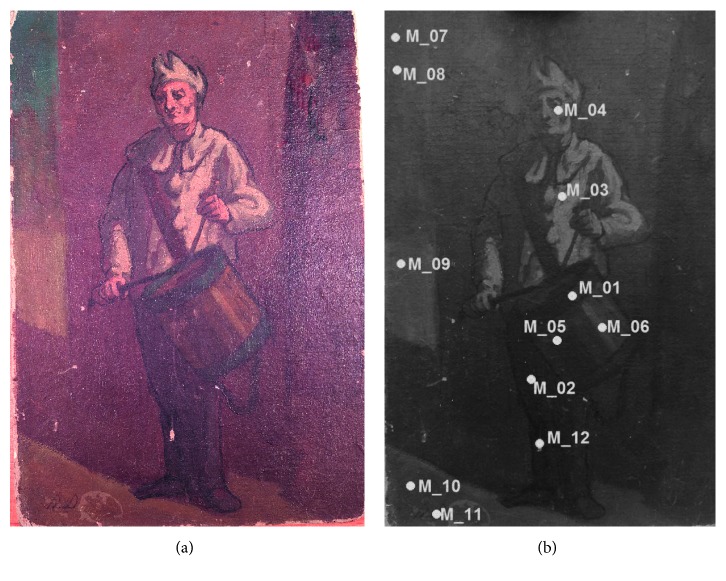
The visible image of the oil painting and the sampling by infrared reflection spectroscopy.

The oil painting on woven paper depicting a “drummer boy” examined is shown in Figure 2. The completely noninvasive study was focused on the characterization of the original materials. Firstly, multispectral imaging techniques, then X-ray fluorescence point by point analyses, and finally reflection infrared spectroscopy were performed.

**Figure 3:**
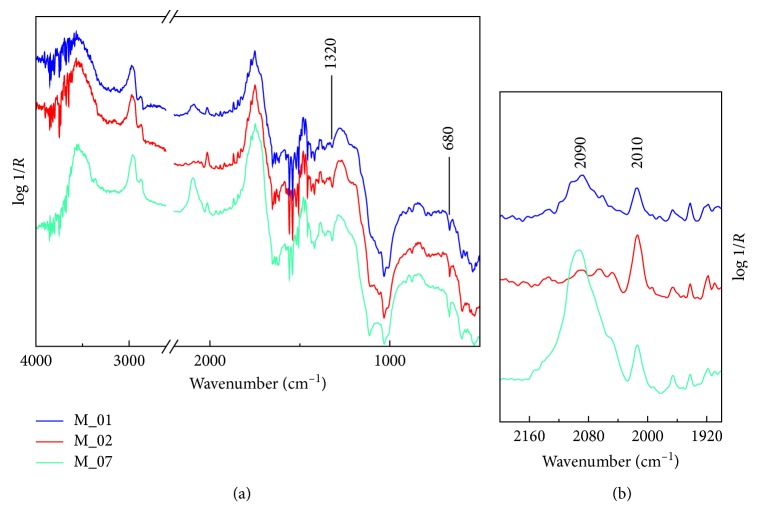
Reflection infrared spectra recorded from the blue hues of the drummer (M_01), from the background (M_08), and from the dark brown area of the musician pant (M_02).

The infrared spectrum profiles recorded from two blue areas (M_01 and M_08) and from a brown area of the musician's trousers (Figure 3) highlight the diagnostic signal of the bone black at the 2013 cm^−1^ in both investigated colors. In blue hue, the bone black pigment is blended with Prussian blue, such as the infrared spectra display the characteristic CN stretching at 2094 cm^−1^ [18]. As mentioned above, the phosphate fundamental bands are strongly distorted by the surface reflection and of little use for diagnostic purposes. The spectra display intense derivative bands at 2920 and 2850 cm^−1^, derivative-like bands at about 1740 and 1460 cm^−1^ ascribable to the lipidic binder, and a signal at 1320 cm^−1^ associated to calcium oxalate [19]. The presence of lead white, as preparation layer, recognizable by the *ν*_4_ at 680 cm^−1^, makes the detection of phosphate bands more challenging.

**Figure 4:**
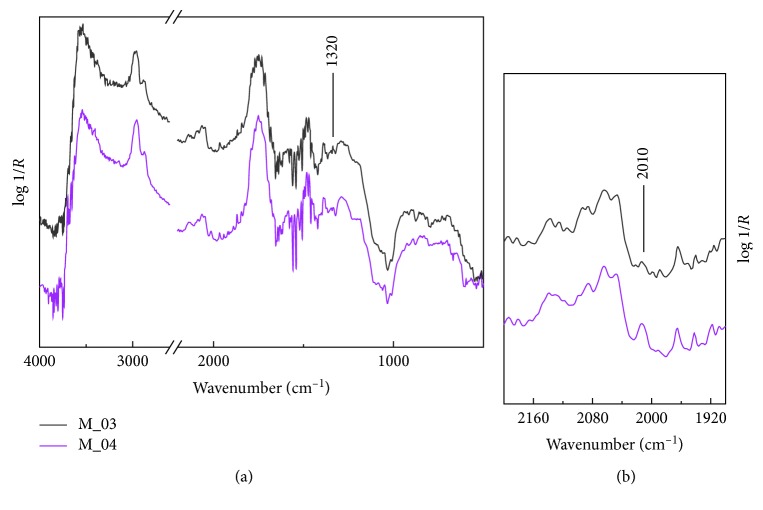
Reflection infrared spectra collected on the grey (M_03) area of the musician dress and on the flesh (M_04).

However, it is important to emphasize that the bone black is still visible even in the presence of barium sulphate (Figure 4) used by the artist as white pigment. Indeed, the infrared spectra recorded on a grey area of the musician's costume and in the flesh clearly exhibit the diagnostic feature at 2013 cm^−1^ of the animal black pigment in spite of the evident combination bands of the sulphate anion in BaSO_4_ (Figure 3(b)) [20].

### 3.2. Mural Painting by Lo Spagna

**Figure 5:**
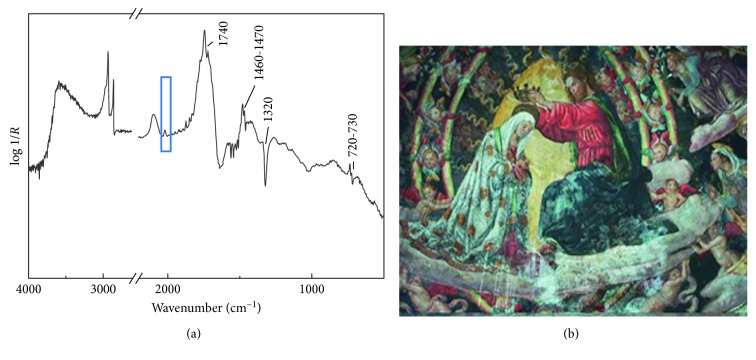
(a) Reflection infrared spectrum collected on a blue sky area of the apse and (b) visible image of the St. Giacomo Church apse.

During the restoration project of the cycle of mural paintings by Lo Spagna in the St. Giacomo Church in Spoleto, a careful diagnostic campaign was planned. The diagnostic support has become necessary for the restorer, especially during the removal of retouching in blue sky and mantle, probably belonging to the three important documented restorations during the nineteenth century. The noninvasive measurements carried out by FTIR spectrometer in the blue areas revealed the presence of azurite, as the original pigment, and Prussian blue as retouching pigment. In Figure 5, the infrared spectrum collected in the apse on a blue hue clearly display the characteristic CN stretching at 2094 cm^−1^ of the Prussian blue [18] and the diagnostic signal of the bone black at the 2013 cm^−1^ band. The spectrum furthermore shows an intense derivative signal at 1320 cm^−1^ characteristic of the CO symmetric stretching of calcium oxalate [19] and intense derivative bands at 2855, 2920, 1740, and derivative-shaped doublets at 1470/1460 and 730/720 cm^−1^ ascribable to a natural wax [21]. The occurrence of oxalate, as degradation products, and of a lipidic component, as retouching materials, hides the fundamental modes of the phosphate group of the bone black pigment.

**Figure 6:**
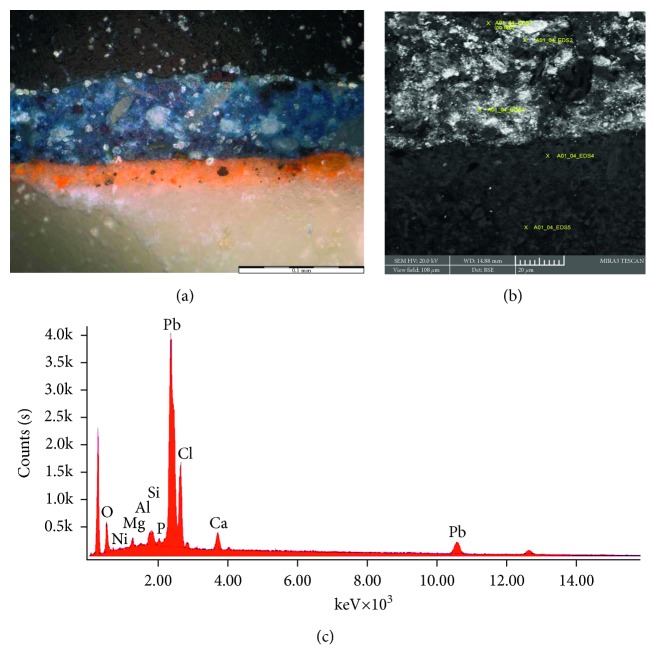
Stratigraphic section of the sample taken on the mural painting. (a) Optical microscope in visible light and (b) the scanning electron microscope showed the points of analysis. (c) EDS spectrum of the blue layer.

Figure 6 shows the cross-sectional optical microscope in visible light and the SEM-BSE images of a sample taken from a blue area in the sky of apse. It is possible to distinguish three different layers: the blue one external, an intermediate one of orange color, and an internal layer of white color ascribable to the plaster. The SEM-EDS analysis collected on the external blue layer showed the occurrence of lead a main element and silicon, calcium, phosphorus, chlorine, and copper as minor components. The presence of phosphorus in the blue layer confirmed the presence of the animal black pigment in mixture with the blue pigment.

These examples show in evidence the utility of the weaker band at 2013 cm^−1^ compared to the fundamental modes that are generally distorted by the surface reflection [17] and fall in spectral range highly affected by possible overlapping with the other constituting materials (binders, pigments, grounds, and degradation products). Despite its usefulness for the identification of an animal black pigment by noninvasive technique, its assignment is still uncertain.

### 3.3. Band Assignment

Vila et al. [13] associate this band probably to a degradation product of the bone compounds produced after the thermal treatment in the synthesis of the black pigment [1, 2]. Tomasini et al. attribute the signal to the cyano group of isocyanate, thiocyanate, and isothyocianate related to degradation products of protein [14]. As reported in literature, the fresh bone infrared spectrum shows only the signals related to organic matter [22, 23].

**Figure 7:**
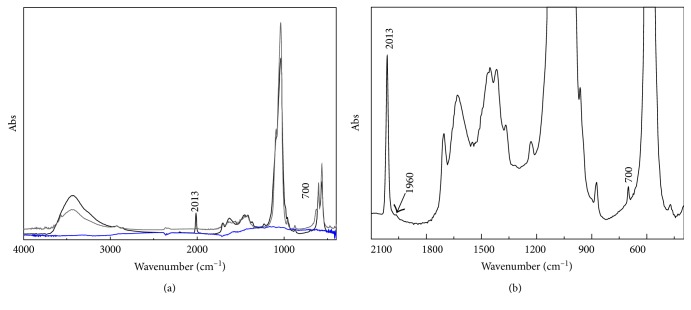
(a) Transmission spectra of vegetal (blue line) and animal black pigments (black line) compared to hydroxyapatite (grey line) standard; (b) enlarged view of the bone black spectrum highlighting the cyanamidapatite characteristic features.

The two commercial animal black pigments, characterized by portable X-ray diffraction, are mainly constituted of hydroxyapatite with impurities of quartz. In Figure 7, the bone black pigment and the hydroxyapatite transmission infrared spectra are compared.

The hydroxyapatite component is clearly underlined in the infrared spectra of bone black pigment commercial sample showing the characteristic phosphate group bands: the *ν*_3_ (PO_4_)^3−^ at 1087 and 1038 cm^−1^, the *ν*_1_ (PO_4_)^3−^ at 875 and 962 cm^−1^, the *ν*_4_ (PO_4_)^3−^ at 630, 604, and 567 cm^−1^, and the *ν*_2_ (PO_4_)^3−^ at 469 cm^−1^ (Figure 7(b)). The IR spectra from the commercial pigments show also a sharp band at 2013 cm^−1^ not present in the hydroxyapatite spectrum (Figure 7(b) and insert) and diagnostic for the identification and distinction of the animal black pigments [13, 14].

Taking into account the wide literature concerning the chemistry of hydroxyapatite, since its important role as inorganic constituent of human bone and teeth, a further assignment for the sharp and distinctive IR band of the bone black pigments at 2013 cm^−1^ may be considered. In detail, Habelitz et al. [24, 25] demonstrated that hydroxyapatite is converted to cyanamidapatite in the presence of dry ammonia and graphite at temperature between 800 and 1300°C. The authors taking into account the results from IR, XRD, and ^31^P NMR suggest that nitrogen enters into the structure as [CN_2_]^2−^ ion, substituting the highly mobility [OH]^−^ groups of hydroxyapatite and forming cyanamidapatite. Their published infrared spectra of the ammonia-treated hydroxyapatite show a sharp band at 2015 cm^−1^ assigned to the *ν*_3_ (CN_2_)^−2^ and a signal at about 700 cm^−1^ associated to the bending mode *ν*_2_. As shown in Figure 7(b), these features are clearly detectable in the IR transmission spectra of the commercial bone blacks. These findings may suggest that a similar reaction could occur when burning the animal bone to produce the black pigment. In fact, the protein constituting the collagen may be the source of nitrogen, whilst the combustion process would provide for carbon favouring the formation of cyano ion substituting the OH groups in the hydroxyapatite structure. To conclude, the sharp band at 2013 cm^−1^, diagnostic for the animal black pigments, is associated with cyanamidapatite, a degradation product of the synthesis of the black pigment.

## 4. Conclusion

Infrared reflection spectroscopy has proven to be a powerful tool for the noninvasive identification of animal carbon black pigments. Two real case studies carried out on different painting materials have been presented in order to highlight the band at 2013 cm^−1^ as marker for the noninvasive identification of animal carbon-based pigments. Previous published data allowed us to suggest a possible assignment of this diagnostic feature. Indeed, the band at 2013 cm^−1^ is associated with the formation of cyanamideapatite during the synthesis of the pigment, involving the bone combustion in the absence of air.
